# Design, synthesis, and evaluation of novel triazole-based carbohydrazide derivatives with notable antioxidant activity: an integrated experimental and DFT study

**DOI:** 10.1186/s13065-026-01736-x

**Published:** 2026-02-23

**Authors:** Nagwan M. Rewish, Mohamed R. Elmorsy, Ahmed A. Fadda, Safa A. Badawy

**Affiliations:** Department of Chemistry, Faculty of Science, Mansoura University, El-Gomhoria Street, Mansoura, 35516 Egypt

**Keywords:** Carbohydrazide, Structural confirmation, Antioxidant activity, DFT, ADME prediction

## Abstract

A series of novel heterocyclic compounds based on 5-methyl-1-(*p*-tolyl)-1*H*-1,2,3-triazole-4-carbohydrazide were designed, synthesized, and evaluated for antioxidant activity. The synthesized derivatives included phthalimide (**NA-1**, **NA-2**), cyanoacetamide (**NA-3**), pyridine-based (**NA-4**), and hydrazonoyl cyanide (**NA-5** to **NA-8**) analogs. Structural confirmation was achieved through IR, NMR (^1^H and ^13^C), and MS analyses. Antioxidant activity was assessed using the DPPH radical-scavenging assay, with ascorbic acid as a reference. Compounds **NA-2** (IC_50_ = 0.072 ± 0.272 mg/mL), **NA-5** (IC_50_ = 0.029 ± 0.135 mg/mL), and **NA-6** (IC_50_ = 0.096 ± 0.085 mg/mL) showed notable activity. DFT calculations suggested favorable electronic properties and hydrogen-bonding potential, supporting their antioxidant behavior. In silico pharmacokinetic analysis (SwissADME) showed acceptable drug-likeness with limited violations in some analogs with Lipinski’s rule of five, indicating acceptable drug-likeness. Bioavailability scores were generally 0.55, except **NA-7** (0.11).

**Supplementary Information:**

The online version contains supplementary material available at 10.1186/s13065-026-01736-x.

## Introduction

**Fig. 1:**
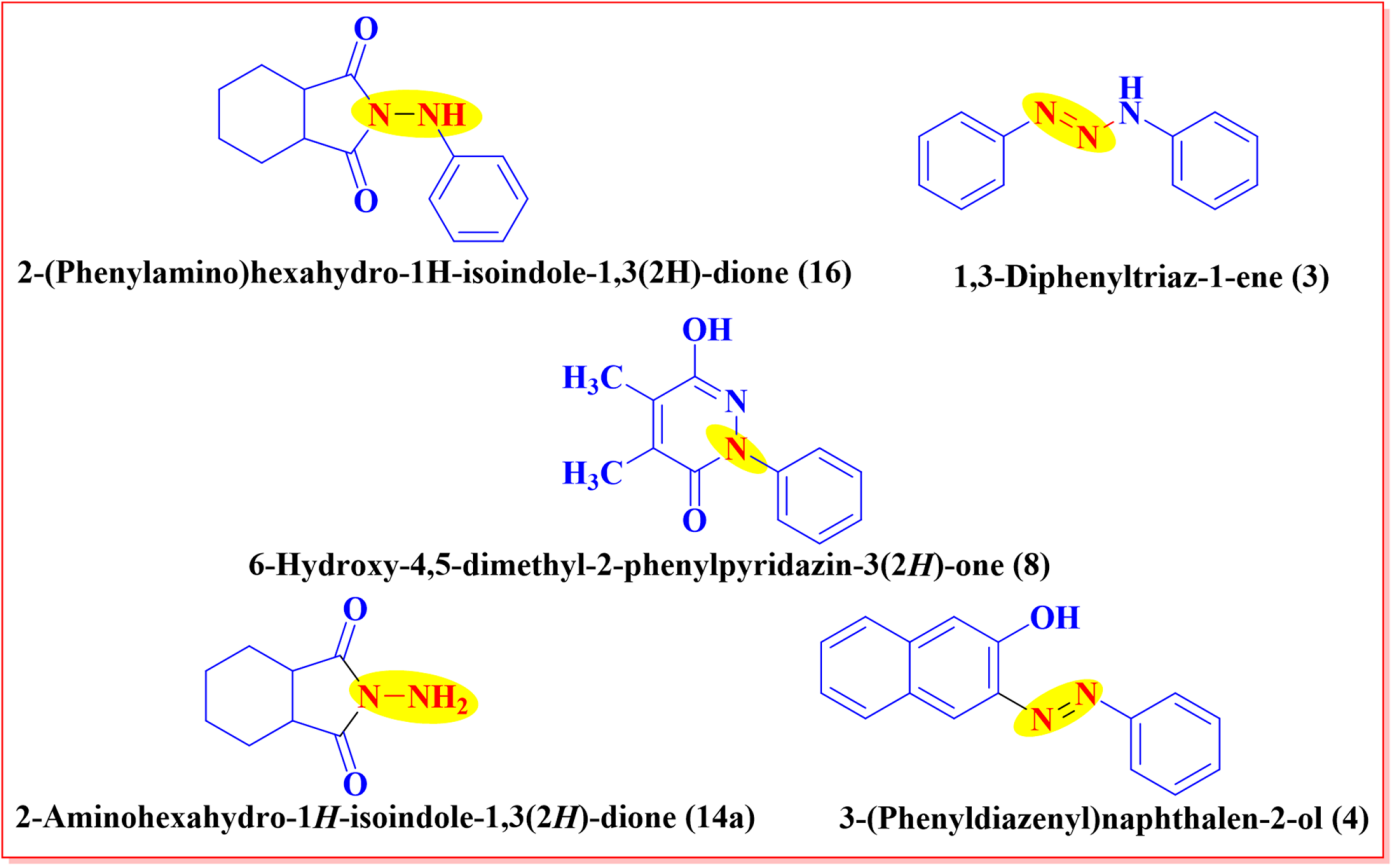
Biologically active compounds with antioxidant properties

Hydrazone-containing compounds have attracted significant interest in medicinal chemistry due to their wide range of pharmacological activities and therapeutic potential, especially in anticancer drug development. Aromatic hydrazones, synthesized by condensing equimolar amounts of aromatic aldehydes with phenolic acid hydrazides under reflux, contain the azomethine (–NH–N=CH–) moiety, a key pharmacophore linked to various biological effects [1]. Hydrazides, a subclass of hydrazine derivatives formed by replacing one or both hydrazine hydrogens with acyl groups (R–CO–), are important in synthetic and pharmaceutical chemistry. Both hydrazides and their hydrazones exhibit diverse biological activities including antiviral, antioxidant, antimicrobial, antimalarial, anti-inflammatory, analgesic, anticancer, antifungal, and antibacterial effects [2, 3]. They are typically synthesized by reacting carboxylic or heterocarboxylic acid hydrazides with aldehydes or ketones in polar protic solvents like ethanol or methanol under reflux [4], and characterized using IR, NMR, and mass spectrometry [5]. The reactive C=N bond in hydrazones is pivotal in forming benzo-fused nitrogen heterocycles, enhancing their medicinal relevance, with many azomethine-containing compounds recently evaluated for biological activity [6]. Concurrently, research over the past decade has emphasized the harmful effects of free radicals, especially the overproduction of reactive oxygen species (ROS), which cause oxidative stress implicated in diseases such as cancer, cardiovascular disorders, neurodegenerative diseases (e.g., Alzheimer’s and Huntington’s), and aging [7]. While free radicals are essential in normal physiology, their excessive accumulation or weakened antioxidant defenses lead to cellular and tissue damage. Antioxidants, whether produced internally or obtained through diet, are vital for maintaining redox balance and preventing oxidative harm. Additionally, free radicals cause oxidative degradation that compromises product quality and shelf life in industries like food, oil, rubber, and petroleum [8, 9]. Consequently, the demand for novel and efficient antioxidants has intensified over the past two decades. Among emerging antioxidant scaffolds, thiol (thiourea)-based heterocyclic compounds have demonstrated remarkable radical-scavenging potential, prompting their extensive investigation [10, 11]. In this context, the synthesis of hydrazone derivatives bearing diverse electron-donating and electron-withdrawing substituents has become a prominent area of research. These structural modifications influence the compounds’ redox behavior, contributing to enhanced antioxidant activity alongside other pharmacological properties. Several hydrazide–hydrazone derivatives have already been successfully translated into therapeutic agents, including iproniazid, nifuroxazide, and isocarboxazid, highlighting their clinical relevance and potential for further drug development [12]. Hydrazide–hydrazone compounds, synthesized through the condensation of hydrazides with aldehydes or ketones, constitute an important class of organic molecules with a diverse pharmacological profile. These compounds have demonstrated a wide range of biological activities, including antimicrobial, antiseptic, antidepressant, antitubercular, antifungal, anti-inflammatory, antiviral, and antiprotozoal properties. Recent studies have also underscored their potential antioxidant activity, further enhancing their significance in medicinal chemistry. The present study was undertaken to design and synthesize novel hydrazide–hydrazone derivatives with potential antioxidant properties, as depicted in Fig. 1. To evaluate their free radical scavenging ability, the DPPH (2,2-diphenyl-1-picrylhydrazyl) assay was employed due to its operational simplicity, rapid execution, cost-effectiveness, and reliability in determining antioxidant capacity [13]. This colorimetric assay is based on the reduction of the stable nitrogen-centered DPPH radical by antioxidants through hydrogen atom donation, resulting in a measurable decrease in absorbance. The absorbance is typically monitored within the 515–528 nm range, corresponding to the loss of the purple color of DPPH upon reduction. For the assay, test compounds were dissolved in methanol to prepare three different concentrations, ranging from 31 to 250 µM. A 1 mL aliquot of each concentration was mixed with an equal volume of 1 mg/mL DPPH solution in methanol. The reaction mixtures were incubated in the dark for 30 min to prevent light-induced degradation. All measurements were conducted in triplicate to ensure reproducibility. The antioxidant activity was determined by calculating the percentage of DPPH radical inhibition. This was achieved by comparing the absorbance of the test sample mixed with DPPH (Abs-sample) to that of the control solution containing only DPPH in methanol (Abs-control). A reduction in absorbance indicated the scavenging of DPPH free radicals by the test compounds. The percentage inhibition was then calculated based on the difference between the control and sample absorbance values, as previously described [14, 15]. While Trolox is frequently employed as a reference standard in antioxidant studies, ascorbic acid was used in this investigation as the positive control for evaluating the antioxidant activity of the synthesized compounds (**NA-1 to NA-8**). Density Functional Theory is a quantum mechanical computational method widely employed to examine the electronic structure of atoms, molecules, and condensed-phase systems. Unlike traditional wavefunction-based approaches, DFT is founded on the electron density as the fundamental variable, rather than the many-body wavefunction. This methodology is underpinned by the Hohenberg–Kohn theorems [16], which establish that all ground-state properties of a many-electron system are uniquely determined by its electron density. Owing to its favorable balance between computational efficiency and accuracy, DFT has become a preferred tool for the prediction and analysis of molecular geometries, electronic distributions, reactivity parameters, and various spectroscopic properties, particularly in large or complex systems [17]. DFT offers indispensable support to experimental work, deepening the understanding of molecular structure, reactivity, and spectroscopy. The approach not only confirms experimental observations but also extends predictability to properties and mechanisms beyond direct measurement [18–20]. The aim of this work was to synthesize and characterize a series of novel carbohydrazide-based compounds (**NA1-8**), investigate their electronic properties using computational methods (such as HOMO-LUMO analysis), and evaluate their antioxidant activities through in vitro DPPH radical scavenging assays. Additionally, the study aimed to assess the pharmacokinetic and drug-likeness profiles of these compounds using in silico ADME prediction tools like SwissADME, to identify promising candidates with potential therapeutic applications and favorable pharmacological properties for further drug development.

## Experimental

General experimental notes: a Gallenkamp device was used to test the melting points, or m.p. The infrared (IR) spectra were estimated using the BRUKER (INVENIO FT-IR spectrometer platform). With magnetic field strengths of 11.75 tesla and BRUKER AvanceCore ^1^H (400 MHz) and ^13^C (100 MHz), ^1^H and ^13^C NMR spectra were acquired at JEOL ECA-500 II ^1^H (500 MHz) and ^13^C (125 MHz) with DMSO-d6 as the solvent. For chemical shifts, the unit of measurement is δ, ppm. The purity of the compounds was confirmed using TLC plates, which were based on a mixture of petroleum ether and ethyl acetate as an eluent. They used a UV lamp as a visual assistant. Utilizing the Termo DSQ II spectrometer at the Faculty of Science at Alazhar University, mass and elemental studies were assessed.

### Synthesis of compounds (NA-1) and (NA-2)

Dissolve 1 mmol of 5-methyl-1-(*p*-tolyl)-1*H*-1,2,3-triazole-4-carbohydrazide (**1**) in 20 mL of glacial acetic acid within a 100 mL round-bottom flask, then add 1 mmol of either phthalic anhydride (**2a**) or 5-nitrophthalic anhydride (**2b**) into the flask. Reflux the reaction mixture for 3 h, then let it cool to room temperature before placing it in an ice bath to promote the precipitation of products (**NA-1**) and (**NA-2**). Recrystallize the product utilizing an appropriate solvent, such as ethanol.

#### N-(1,3-Dioxoisoindolin-2-yl)-5-methyl-1-(p-tolyl)-1 H-1,2,3-triazole-4-carboxamide (NA-1)

White crystal (82% yield); m.p. above 300 °C. IR (ῡ, cm^− 1^): 3215 (N–H), 1741, 1676 (C=O). ^1^H NMR (DMSO-*d*_6_): *δ* (ppm): 2.41 (s, 3 H, –CH_3_), 2.48 (s, 3 H, –CH_3_), 7.44 (d, *J* = 8.00 Hz, 2 H, Ar–H), 7.54 (d, *J* = 8.50 Hz, 2 H, Ar–H), 7.96 (q, *J* = 3.00 Hz, 2 H, Ar–H), 8.01 (q, *J* = 3.00 Hz, 2 H, Ar-H), 11.41 ( s, 1H, N–H). ^13^C NMR (DMSO-*d*_6_): *δ* (ppm): 9.3, 20.8, 123.9 (2 C), 125.4 (2 C), 129.4 (2 C), 130.1 (2 C), 130.2, 132.6, 135.5 (2 C), 135.5, 138.7, 159.9, 165.2 (2 C). Mass analysis (m/z, %): 361 (M^+,^ 34.84), 345 (80.42), 340 (87.83), 337 (100.00), 330 (73.01), 351 (54.85), 250 (57.38), 243 (70.04), 240 (59.79), 238 (71.46), 211 (71.59), 157 (58.25), 150 (54.23), 144 (59.30), 129 (75.73). Analysis for: C_19_H_15_N_5_O_3_ (361.12): Calculated: C, 63.15; H, 4.18; N, 19.38. Found: C, 63.25; H, 4.19; N, 19.43%.

#### 5-Methyl-N-(5-nitro-1,3-dioxoisoindolin-2-yl)-1-(p-tolyl)-1 H-1,2,3-triazole-4-carboxamide (NA-2)

White powder (80% yield); m.p. above 300 °C. IR (ῡ, cm^− 1^): 3236 (N–H), 1748, 1690 (C=O). ^1^H NMR (DMSO-*d*_6_): *δ* (ppm): 1.92 (s, 3 H, –CH_3_), 2.49 (s, 3 H, –CH_3_), 7.48 (d, *J* = 8.00 Hz, 2 H, Ar–H), 7.57 (d, *J* = 8.40 Hz, 2 H, Ar–H), 8.30 (d, *J* = 8.40 Hz, 1H, Ar–), 8.67 (d, *J* = 1.60 Hz, 1H, Ar–H), 8.76 (q, *J* = 2.00 Hz, 1H, Ar–H), 11.64 ( s, 1H, N–H). Mass analysis (m/z, %): 406 (M^+,^ 29.39), 400 (28.15), 370 (25.54), 306 (62.98), 267 (70.76), 263 (33.63), 207 (31.77), 181 (19.45), 169 (80.23), 159 (63.07), 112 (40.68), 68 (54.45), 65 (56.10), 43 (100.00). Analysis for: C_19_H_14_N_6_O_5_ (406.10): Calculated: C, 56.16; H, 3.47; N, 20.68. Found: C, 56.24; H, 3.46; N, 20.70%.

#### Synthesis of N’-(2-cyanoacetyl)-5-methyl-1-(p-tolyl)-1 H-1,2,3-triazole-4-carbohydrazide (NA-3)

A 50 mL round-bottom flask was desiccated, and 30 mL of anhydrous dioxane was used to dissolve 0.23 g (1 mmol) of 5-methyl-1-(*p*-tolyl)-1*H*-1,2,3-triazole-4-carbohydrazide (**1**) and 0.16 g (1 mmol) of newly produced 3-(3,5-dimethyl-1*H*-pyrazol-1-yl)-3-oxopropanenitrile (**3**). Three hours were spent allowing the reaction mixture to reflux. The off-white precipitate was produced and subsequently isolated via filtration after the solution was allowed to cool to ambient temperature, resulting in the formation of compound (**NA-3**). A pale powder was obtained with a yield of 70%, and its melting point was recorded at 178–180 °C [21].

Off white (75% yield), m.p. 178–180 °C. IR (ῡ, cm^− 1^): 3228 (N–H), 3038 (=C–H), 2933 (C–H) aliphatic, 2258 (C ≡N), 1702 and 1665 (C=O). ^1^H NMR (DMSO-*d*_6_): *δ* (ppm): 2.41 (s, 3 H, –CH_3_), 2.48 (s, 3 H, –CH_3_), 3.79 (s, 2 H, –CH_2_), 7.43 (d, *J* = 8.00 Hz, 2 H, Ar–H), 7.50 (d, *J* = 8.00 Hz, 2 H, Ar-H), 10.29 (s, 1H, –NH), 10.59 (s, 1H, -NH). ^13^C NMR (DMSO-*d*_6_): *δ* (ppm): 9.3, 20.8, 23.9, 115.7, 125.3 (2 C), 130.1 (2 C), 132.8, 136.4, 137.7, 139.9, 159.9, 161.8. Mass analysis (m/z, %): 298 (M^+,^ 28.42), 296 (24.21), 294 (23.91), 286 (50.49), 284 (53.67), 280 (26.24), 241 (46.38), 222 (28.93), 206 (60.79), 203 (26.01), 172 (36.80),166 (70.42), 137 (100.00), 102 (31.95). Analysis for: C_14_H_14_N_6_O_2_ (298.12): Calculated: C, 56.37; H, 4.73; N, 28.17. Found: C, 56.47; H, 4.74; N, 28.20%.

### Synthesis of N-(3-cyano-4,6-dimethyl-2-oxopyridin-1(2 H)-yl)-5-methyl-1-(p-tolyl)-1 H-1,2,3-triazole-4-carboxamide (NA-4)

Compound (**NA-3**) (1 mmol) and acetylacetone (**4**) (1 mmol) were combined in ethanol (15 mL), and a few drops of piperidine (3 drops) were added to the mixture. The reaction was refluxed for ten hours. Following the cooling of the reaction mixture, the solid that was formed was filtered out and then recrystallized from ethanol to produce (**NA-4**).

Yellow powder (80% yield); m.p. above 300 °C. IR (ῡ, cm^− 1^): 3275 (N–H), 2223 (C≡ N), 1701, 1665 (C=O). ^1^H NMR (DMSO-*d*_6_): *δ* (ppm): 2.28 (s, 3 H, –CH_3_), 2.37 (s, 3 H, –CH_3_), 2.41 (s, 3 H, –CH_3_), 2.49 (s, 3 H, -CH_3_), 6.42 (s, 1H, Ar-H), 7.43 (d, *J* = 8.00 Hz, 2 H, Ar–H), 7.53 (d, *J* = 8.00 Hz, 2 H, Ar-H), 11.64 (s, 1H, N–H). ^13^C NMR (DMSO-*d*_6_): *δ* (ppm): 9.3, 18.9, 20.8, 20.8, 100.1, 108.3, 115.5, 125.3(2 C), 130.2(2 C), 132.6, 135.6, 138.8, 140.1, 154.7, 158.2, 159.9, 160.2. Mass analysis (m/z, %): 362 (M^+,^ 25.73), 356 (29.63), 344 (29.83), 341 (32.63), 319 (41.55), 315 (47.57), 260 (100.00), 247 (38.34), 233 (26.37), 222 (36.01), 221 (50.56), 217 (45.81), 210 (30.13), 205 (37.00), 201 (34.55). Analysis for: C_19_H_18_N_6_O_2_ (362.15): Calculated: C, 62.97; H, 5.01; N, 23.19. Found: C, 63.05; H, 5.00; N, 23.22%.

### Synthesis of the derivative NA-5-8

A 0.3 ml concentrated HCl solution containing *p*-substituted aniline (**5a-d**) (1 mmol) was stirred and chilled to 0–5 °C. The chilled mixture was subjected to diazotization by the introduction of a NaNO_2_ solution (0.07 g in 5 ml H_2_O) [22]. The fresh diazonium chloride solution was meticulously added dropwise to a chilled suspension of *N*’-(2-cyanoacetyl)-5-methyl-1-(*p*-tolyl)-1*H*-1,2,3-triazole-4-carbohydrazide (**NA-3**) (1 mmol) in pyridine (15 ml). The solid product was collected through filtration, and stirring was maintained at 0–5 °C for an additional hour. Compounds **NA-5-8** was produced as a result of the recrystallization of the collected solid in ethanol.

#### N-(4-Methoxyphenyl)-2-(2-(5-methyl-1-(p-tolyl)-1 H-1,2,3-triazole-4-carbonyl)hydrazineyl)-2-oxoacetohydrazonoyl cyanide (NA-5)

Yellow powder (75% yield); m.*p* = 230–232 °C. IR (ῡ, cm^− 1^): 3356, 3287, 3232(N-H), 2214 (C ≡ N), 1656 (C=O). ^1^H NMR (DMSO-*d*_6_): *δ* (ppm): 2.41 (s, 3 H, –CH_3_), 2.49 (s, 3 H, –CH_3_), 3.75 (s, 3 H, –CH3), 6.97 (q, *J* = 9.00 Hz, 2 H, Ar–H), 7.41 (d, *J* = 9.50 Hz, 1H, Ar-H), 7.44 (d, *J* = 8.00 Hz, 2 H, Ar–H), 7.52 (d, *J*=8.00 Hz, 2 H, Ar-H), 7.69 (d, *J* = 9.00 Hz, 1H, Ar–H), 10.20 ( s, 1H, N–H). 10.47 (s, 1H, N–H), 11.90 (s, 1H, N–H). ^13^C NMR (DMSO-*d*_6_): *δ* (ppm): 9.3, 20.8, 55.3, 104.6, 111.4, 114.4(2 C), 117.6(2 C), 125.2(2 C), 130.1(2 C), 132.8, 135.6, 136.8, 137.5, 139.9, 156.5, 160.4, 160.7. Mass analysis (m/z, %): 432 (M^+,^ 11.68), 413 (36.30), 391 (16.37), 382 (18.75), 381 (40.88), 367 (50.68), 338 (28.69), 313 (26.40), 302 (34.70), 286 (65.67), 223 (48.77), 215(36.30), 209 (29.04), 186 (18.58), 110 (100.00). Analysis for: C_21_H_20_N_8_O_3_ (432.17): Calculated: C, 58.33; H, 4.66; N, 25.91. Found: C, 58.40; H, 4.65; N, 25.97%.

#### N-(4-Chlorophenyl)-2-(2-(5-methyl-1-(p-tolyl)-1 H-1,2,3-triazole-4-carbonyl)hydrazineyl)-2-oxoacetohydrazonoyl cyanide (NA-6)

Yellow powder (83% yield); m.*p*=235–238 °C. IR (ῡ, cm^− 1^): 3368, 3293, 3227 (N-H), 2221 (C ≡ N), 1678 (C=O). ^1^H NMR (DMSO-*d*_6_): *δ* (ppm): 2.38 (s, 3 H, –CH_3_), 2.45 (s, 3 H, –CH_3_), 7.38 (s, 1H, Ar–H), 7.40 (d, *J* = 7.50 Hz, 2 H, Ar–H), 7.45 (s, 1H, Ar–H), 7.49 (d, *J* = 8.00 Hz, 2 H, Ar–H), 7.74 (d, *J* = 9.00 Hz, 2 H, Ar-H), 10.31 ( s, 1H, N–H), 10.49 (s, 1H, N–H), 10.68 (s, 1H, N–H). ^13^C NMR (DMSO-*d*_6_): *δ* (ppm): 9.7, 21.2, 107.4, 111.4, 118.3(2 C), 125.7(2 C), 128.6, 129.54(2 C), 130.6(2 C), 133.3, 137.2, 138.0, 140.4, 141.5, 160.7, 160.8. Mass analysis (m/z, %): 436 (M^+,^ 49.04), 348 (45.68), 346 (50.46), 314 (40.40), 285 (50.63), 274 (44.09), 238 (100.00), 220 (59.01), 195 (62.52), 183 (73.08), 151 (40.84), 134(49.96), 129 (48.89), 120 (55.00). Analysis for: C_20_H_17_ClN_8_O_2_ (436.12): Calculated: C, 54.99; H, 3.92; N, 25.65. Found C, 55.05; H, 3.93; N, 25.68.%.

#### 4-(2-(1-Cyano-2-(2-(5-methyl-1-(p-tolyl)-1 H-1,2,3-triazole-4-carbonyl)hydrazineyl)-2-oxoethylidene)hydrazineyl)benzoic acid (NA-7)

Yellow powder (79% yield); m.p. above 300 °C. IR (ῡ, cm^− 1^): 3361, 3306, 3241 (N–H), 2218 (C ≡ N), 1714, 1676 (C=O). ^1^H NMR (DMSO-*d*_6_): *δ* (ppm): 2.41 (s, 3 H, –CH_3_), 2.49 (s, 3 H, –CH_3_), 7.44 (d, *J* = 8.00 Hz, 2 H, Ar–H), 7.53 (d, *J* = 8.00 Hz, 2 H, Ar-H), 7.83 (d, *J* = 9.00 Hz, 2 H, Ar-H), 7.92 (d, *J* = 9.00 Hz, 2 H, Ar–H), 10.40 (s, 1H, N–H), 10.56 ( s, 1H, N–H), 12.17 (s, 1H, N–H), 12.83 (s, 1H, –COOH). ^13^C NMR (DMSO-*d*_6_): *δ* (ppm): 9.3, 20.8, 108.1, 110.8, 115.8(2 C), 125.3(2 C), 126.0, 130.1(2 C), 130.6(2 C), 132.8, 136.7, 137.6, 139.9, 145.7, 160.1, 160.3, 166.9. Mass analysis (m/z, %): 446 (M^+,^ 59.94), 443 (67.77), 442 (60.24), 437 (60.19), 429 (62.95), 417 (100.00), 416 (49.35), 411 (40.66), 364 (42.67), 360 (42.37), 339 (43.22), 315(44.68), 309 (59.59), 278 (44.58), 229 (44.78). Analysis for: C_21_H_18_N_8_O_4_ (446.15): Calculated: C, 56.50; H, 4.06; N, 25.10. Found: C, 56.59; H, 4.05; N, 25.15%.

#### 2-(2-(5-Methyl-1-(p-tolyl)-1 H-1,2,3-triazole-4-carbonyl)hydrazineyl)-N-(4-nitrophenyl)-2-oxoacetohydrazonoyl cyanide (NA-8)

Yellow powder (81% yield); m.*p* = 267–269 °C. IR (ῡ, cm^− 1^): 3370, 3317, 3214 (N–H), 2223 (C ≡ N), 1665 (C=O). ^1^H NMR (DMSO-*d*_6_): *δ* (ppm): 2.44 (s, 3 H, –CH_3_), 2.52 (s, 3 H, –CH_3_), 7.47 (d, *J*= 8.00 Hz, 2 H, Ar-H), 7.56 (d, *J* = 8.00 Hz, 2 H, Ar-H), 7.97 (d, *J* = 9.20 Hz, 2 H, Ar-H), 8.27 (d, *J* = 8.80 Hz, 2 H, Ar-H), 10.56 (s, 1H, N–H), 10.64 ( s, 1H, N–H), 12.39 (s, 1H, N–H). Mass analysis (m/z, %): 447(M^+,^ 41.09), 380 (24.43), 349 (28.55), 335 (22.53), 321 (30.69), 298 (58.24), 217 (28.52), 211 (29.33), 167 (34.41), 156 (26.46), 150 (29.69), 144 (40.04), 141 (100.00), 87 (34.90), 80 (34.02). Analysis for: C_20_H_17_N_9_O_4_ (447.14): Calculated: C, 53.69; H, 3.83; N, 28.18. Found: C, 53.75; H, 3.82; N, 28.24; %.

### Calculations for quantum chemicals: global reactivity analysis and geometry optimization

The energetic geometry of all compounds was reduced and saved in a (*.mol) file format using the Chem 3D 16.0 software. The Gaussian 09 W program handles the compounds’ geometry optimizations (https://gaussian.com/) [23]. The DFT computations were performed using Becke’s three-parameter hybrid exchange-correlation functional (B3LYP) at the 6-311 + + G(d, p) basis set [24]. We use the B3LYP/6-311 + + G(d, p) to calculate total energies, band gaps, frontier molecular orbital energies (HOMOs and LUMOs), and chemical properties such dipole moments (µ), electronegativity (χ), hardness (η), softness (σ) and nucleophilicity (*Nu*). B3LYP is a hybrid DFT functional that combines the Lee-Yang-Parr correlation function and Becke’s 3-parameter exchange function. The dependability, speed, and precision of organic molecules were balanced [25]. Compared to minimal or double-zeta basis sets (e.g., STO-3G, 6-31G), the split-valence triple-zeta basis set 6-311G is more precise [26]. Adding polarization functions (d for heavy atoms and p for hydrogens) improves geometry and bonding accuracy [27]. (++) adds diffuse functions to heavy atoms and hydrogens, which are essential for long-range charge dispersion, anions, lone pairs, and hydrogen bonding [28]. For the reasons stated above, the B3LYP/6-311 + + G(d, p) basis sets are ideal for DFT computations of organic compounds [29].

### Antioxidant activity using DPPH assay

A serial dilution of each sample was prepared by mixing the sample solution with an equivalent volume of methanol. A 0.135 mg/mL DPPH solution was prepared and combined with an equivalent volume of each concentration from the serial dilution. 1 mL of DPPH^•^ solution was added to each tube, and the tubes were incubated at room temperature in the dark for 30 min. The absorbance was measured at 517 nm. The following equation (Eq. 1) was used to calculate the % of remaining DPPH [30]:

1 $$\:\%\:\mathrm{D}\mathrm{P}\mathrm{P}\mathrm{H}^{\cdot}\:\:\mathrm{r}\mathrm{e}\mathrm{m}\mathrm{a}\mathrm{i}\mathrm{n}\mathrm{i}\mathrm{n}\mathrm{g}=\frac{\left[\mathrm{D}\mathrm{P}\mathrm{P}\mathrm{H}^{\cdot}\right]\:\mathrm{T}}{\left[\mathrm{D}\mathrm{P}\mathrm{P}\mathrm{H}^{\cdot}\right]\mathrm{T}=0}\:\mathrm{x}\:100$$

The values were calculated applying an exponential curve plotting the % of remaining DPPH^•^ versus the sample concentration in mg/mL. The IC_50_ denotes the quantity of antioxidants necessary to diminish the initial concentration of the DPPH solution by 50%. The IC_50_ values demonstrate an inverse relationship with the antioxidant capability of the examined sample [31].

### Estimation of the pharmacokinetic and drug likeness properties

Initially, the compounds’ chemical 2D structures were created using Marvin Sketch (ChemAxon, Version 18.30). The abbreviation for the process representing Absorption, Distribution, Metabolism, and Excretion is ADME. The SwissADME tool’s online SMILES translator was then used to transform the drawings into SMILES format. The Swiss Institute of Bioinformatics developed the web program SwissADME to predict the pharmacokinetic properties and drug-likeness of the synthesized substances. Additionally, the compounds’ SMILES representations were uploaded to the web-based AdmetSAR-2.0 platform for a comprehensive analysis. This combined computational approach offers a reliable early-stage evaluation of the compounds’ pharmacokinetic profiles and safety characteristics for medication development. By using a number of methods, the study increases confidence in the predictions about the compounds’ potential toxicity as well as their absorption, distribution, metabolization, and elimination processes. This information helps prioritize compounds with beneficial ADMET characteristics before proceeding with costly and time-consuming experimental testing [32].

## Results and discussions

### Synthesis of carbohydrazide-based compounds (NA1-8)

**Scheme 1:**
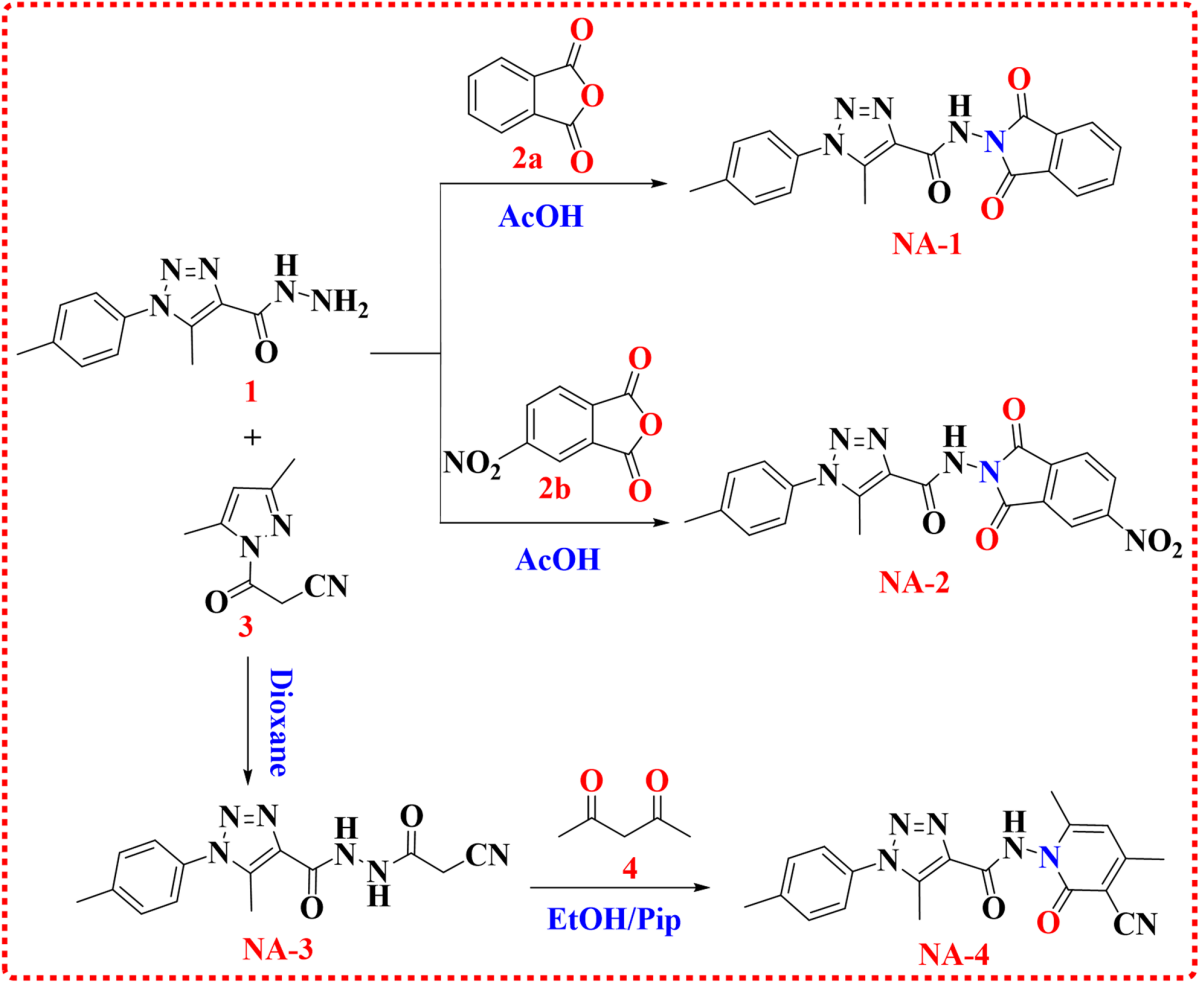
Synthesis of phthalimide derivatives (**NA-1**,** NA-2**) and pyridone derivatives (**NA-4**)

The synthesis of *N*-(1,3-dioxoisoindolin-2-yl-5-methyl-1-(*p*-tolyl)-1*H*-1,2,3-triazole-4-carboxamide (**NA-1**) and 5-methyl-*N*-(5-nitro-1,3-dioxoisoindolin-2-yl-1-(*p*-tolyl)-1*H*-1,2,3-triazole-4-carboxamide (**NA-2**) was synthesized by refluxing 5-methyl-1-(*p*-tolyl)-1*H*-1,2,3-triazole-4-carbohydrazide (**1**) in glacial acetic acid with phthalic anhydride (**2a**) or 5-nitrophthalic anhydride (**2b**). Both reactions produced the target compounds in high yields (82% for **NA-1** and 80% for **NA-2**), demonstrating the effectiveness of this synthetic method. In compound **NA-1**, the spectrum displays two singlets at 2.41 and 2.48 ppm, each integrating for three protons, corresponding to two non-equivalent aromatic methyl groups. A pair of doublets at 7.44 (*J* = 8.0 Hz, 2 H) and 7.54 ppm (*J* = 8.5 Hz, 2 H) are attributed to aromatic protons. Additional aromatic signals at 7.96 and 8.01 ppm appear as quartets (*J* = 3.0 Hz, 2 H). A highly deshielded singlet at 11.41 ppm corresponds to the amide N–H proton. Signals at 9.3 and 20.8 ppm confirm the presence of two chemically distinct methyl carbons. Multiple resonances between 123.9 and 138.7 ppm correspond to aromatic carbons, two highly deshielded signals at 165.2 ppm (2 C) are consistent with conjugated carbonyl carbons, supporting the presence of amide functionalities. The nitro phthalimide ring in **(NA-2)** has three aromatic protons attached to the p-tolyl group, the protons’ relative abundances are 8.30, 8.67 and 8.76 ppm. The preparation of compound (**NA-4**) started with the synthesis of the crucial intermediate, *N*’-(2-cyanoacetyl)-5-methyl-1-(*p*-tolyl)-1*H*-1,2,3-triazole-4-carbohydrazide (**NA-3**). This compound was obtained by reacting 5-methyl-1-(*p*-tolyl)-1*H*-1,2,3-triazole-4-carbohydrazide (**1**) with 3,5-dimethyl-1-cyanoacetylpyrazole (**3**) in dioxane solvent. The compound (**NA-4)** derivative was produced via the reaction of Compound **NA-3** with acetylacetone (**4**) as show in Scheme 1 [33]. The presence of three absorption bands at 3275, 2223, 1716 and 1661 cm^− 1^ in the IR spectra was attributed to the presence of –NH, CN and two CO groups. The ^1^H NMR spectrum showed four methyl singlets, with one methyl on the triazole ring (C5), two methyl’s on the pyridone ring (C4, C6), and one methyl on the *p*-tolyl ring (para position), as confirmed by the δ values of 2.28, 2.37, 2.41 and 2.49 ppm Hydrogen bonding causes a downfield shift of 11.64 ppm for the amide N–H. There are notable carbon signals at δ 9.38 ppm, 18.93, 20.84 and 20.87 ppm for methyl carbons. The predicted molecular weight of C_19_H_18_N_6_O_2_ is matched by the molecular ion peak at m/z 362 (M⁺, 25.73%).

**Scheme 2:**
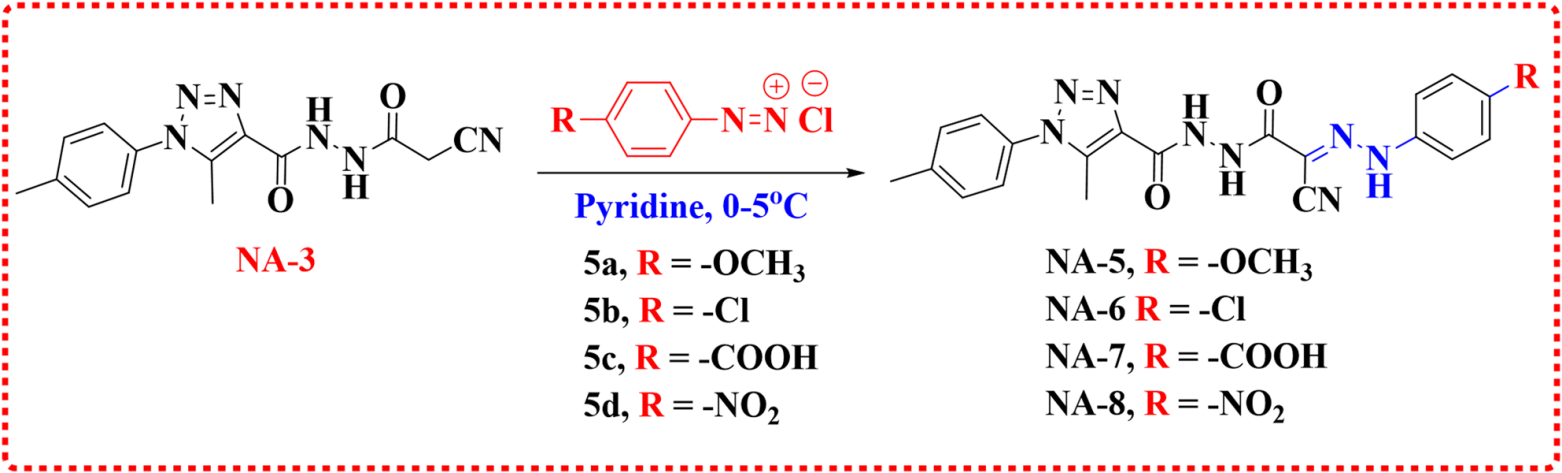
Synthesis of 1,2,3-Triazole-based acylhydrazone–cyanohydrazonoyl derivatives (**NA-5-8**)

The methylene group in the cyanoacetamide derivative (**NA-3**) that was created during electrophilic coupling reactions with aryl diazonium chlorides (**5a-d**). More precisely, at temperatures between 0 and 5 °C, it was mixed with other diazotized anilines, including 4-anisidine, 4- chloroaniline, 4-aminobenzoic acid, and 4-nitroaniline, in pyridine. This resulted in the production of the corresponding hydrazone derivatives (**NA-5-8)** (Scheme 2) [34]. The chemical structures of (**NA-5-8**) were examined and confirmed using elemental and spectroscopic examinations. The IR spectra of **NA-5** showed the presence of 3(–NH), CN, and CO groups at (3356,3287, 3232), 2214, and 1656 cm^− 1^ ,respectively. **NA-5**, with δ values of 3.75 ppm, supports the presence and substitution pattern of the aromatic moiety, attributing it to the 4-methoxyphenyl ring’s methoxy (–OCH_3_) group. Three distinct NH protons, with δ values of 10.20, 10.47, and 11.90 ppm, indicate the amide group, hydrazone, and hydrazide, respectively. The carbons of the amide carbonyl (C=O) and carbon (C=N) are responsible for the alterations between 156 and 160 ppm. The molecular ion peak (M⁺) at m/z=432 (11.68%) corresponds to the exact molecular weight of C_21_H_20_N_8_O_3_, supporting the proposed structure. Compound **NA-6** has numerous aromatic protons signatures and has a δ range of 7.38–7.74 ppm. The methyl carbons on the triazole ring and p-tolyl group have masses of 9.79 and 21.28 ppm, respectively. These high ppm values, 160.76 and 160.85 ppm, correspond to the carbonyl carbon (C=O) and carbonyl nitrogen (C=N) groups. The carbonyl of the carboxylic acid group appears at 1714 cm^− 1^ in the infrared spectrum of **NA-7**. The existence of a methyl group on the triazole ring at the 5-position and a methyl group on the p-tolyl ring is confirmed by two singlets (2.41 & 2.49 ppm) in **NA-7**, which integrate to three protons. The aromatic protons of the benzoic acid ring are responsible for two para-substituted doublets with two hydrogen atoms each at 7.83 and 7.92 ppm. A carboxylic acid (COOH) containing a singlet hydrogen atom has a proton at 12.83 parts per million. The COOH carbon at about 167 ppm further confirms the substitution with benzoic acid. The molecular ion peak (M⁺) at m/z=446 (59.94%) exactly corresponds to the molecular weight of C_21_H_18_N_8_O_4_ (446.15 g/mol). The existence of two protons is indicated by the two doublets at δ 7.97 and 8.27 ppm for **NA-8** that are present in para-substituted aromatic systems. 4-nitrophenyl groups are characterized by these signals. The δ values of 10.56, 10.64, and 12.39 ppm most likely correspond to the amide, hydrazone, and hydrazide. The structures of all products NA1–8 were confirmed, as shown in Figures S1–S30 of the Supplementary Information.

### Frontier molecular orbital study of carbohydrazide derivatives

Frontier molecular orbitals (FMOs), in particular the highest occupied (HOMO) and lowest unoccupied (LUMO) molecular orbitals, have been widely used by chemists to anticipate and study the reactivity and regioselectivity of a variety of chemical systems [35] The structures of carbohydrazide derivatives were synthesized using DFT by Gauss View 6.0 and the Gaussian 09 W software program as shown in Eqs. 2–7 [36]. Table 1 presents the computed global reactivity descriptors, including electronegativity (χ), chemical potential (µ), global hardness (η), electrophilicity index (ω), global softness (σ), and nucleophilicity index (Nu), all derived according to Eqs. (2–7) [37]– [38].

2 $$\:\chi\:=-\frac{1}{2}\mathrm{(}{E}_{HOMO}\mathrm{+}{E}_{LUMO}\mathrm{)}$$

3 $$\:\mu\:=-\chi\:=\frac{1}{2}\mathrm{(}{E}_{HOMO}\mathrm{+}{E}_{LUMO}\mathrm{)}$$

4 $$\:\eta=\frac{1}{2}\mathrm{(}{E}_{HOMO}\mathrm{-}{E}_{LUMO}\mathrm{)}$$

5 $$\:\omega\:=\frac{\mu\:2}{2\upeta}$$

6 $$\:\sigma\:=\frac{1}{2\upeta}$$

7 $$\:Nu=\frac{1}{\omega\:}$$

When a molecule has high antioxidant activity, it is often associated with a lower band gap energy between its HOMO and LUMO [37, 39]. The orbitals of HOMO and LUMO molecules were used to determine the distribution of their wavefunctions and relative reactivity. Table 1 demonstrates how altering the substituents altered the compounds’ band gap energies and reactivity [38]. A small HOMO-LUMO gap refers to a soft molecule with low chemical hardness, which has increased nucleophilicity and chemical reactivity. In contrast, a large gap indicates a rigid molecule with a low nucleophilic potential and improved stability [40].

**Table 1:** The produced substances’ quantum chemical descriptors for the compounds **(NA-1-8)**

Compound	E_H_ _(eV)_	E_L_ _(eV)_	E_g_ _(eV)_	Χ _(eV)_	µ _(eV)_	η _(eV)_	ω _(eV)_	σ _(eV_^−1^_)_	Nu _(eV)_
**NA-1**	− 6.11	− 2.44	3.67	4.27	-4.27	1.83	4.98	0.27	0.20
**NA-2**	− 6.43	− 3.47	2.96	4.95	-4.95	1.48	8.27	0.33	0.12
**NA-3**	− 5.74	− 2.27	3.47	4.00	-4.00	1.73	4.61	0.28	0.21
**NA-4**	− 3.69	− 2.22	1.17	2.95	-2.95	0.73	5.96	0.68	0.16
**NA-5**	− 6.15	− 2.87	3.28	4.51	-4.51	1.64	6.20	0.30	0.16
**NA-6**	− 5.43	− 3.22	2.21	4.32	-4.32	1.10	8.48	0.45	0.11
**NA-7**	− 6.23	− 3.19	3.04	4.71	-4.71	1.52	7.29	0.32	0.13
**NA-8**	− 6.58	− 3.69	2.89	5.13	-5.13	1.44	9.13	0.34	0.10

**Fig. 2:**
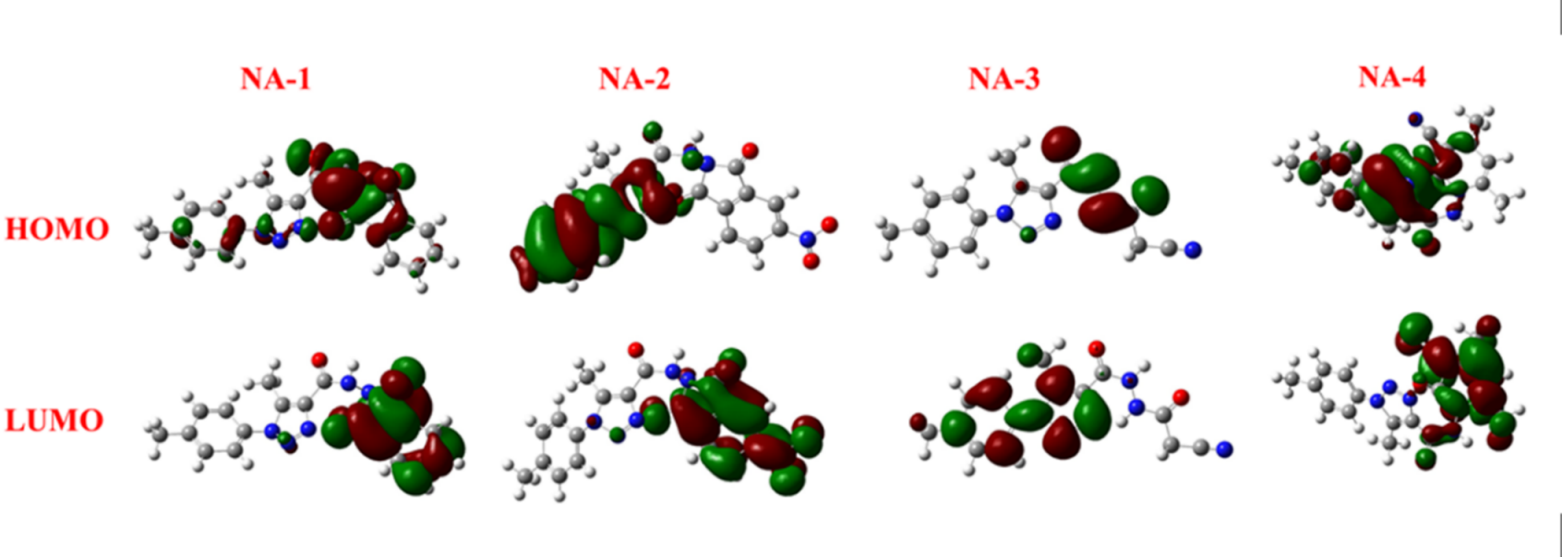
HOMOs and LUMOs geometries for compounds **NA-1-4**

As shown in Fig. 2, this basically trivial structural alteration results in a substantial modification of their electrical characteristics. The global hardness (η) is an essential parameter for understanding electron-donating capabilities relevant to antioxidant behavior. Molecules with lower hardness values are softer and can more easily donate electrons, enhancing their antioxidant capacity. **NA-5** stands out with good antioxidant activity and moderate hardness (η=1.64 eV). This suggests an optimal balance between electronic stability and reactivity. **NA-6** and **NA-2**, with relatively low hardness values (1.10 and 1.48 eV, respectively), also exhibit strong antioxidant properties (low IC_50_). **NA-1** and **NA-3**, being harder molecules (η > 1.7 eV), display weaker antioxidant activities, supporting the general trend. The chemical potential (µ), which reflects the tendency of a molecule to donate electrons and describes the escaping tendency of electrons from a system at equilibrium, follows the decreasing order: **NA-8** (− 5.13 eV) > **NA-2** (− 4.95 eV) > **NA-7** (− 4.71 eV) > **NA-5** (− 4.51 eV) > **NA-6** (− 4.32 eV) > **NA-1** (− 4.27 eV) > **NA-3** (− 4.00 eV) > **NA-4** (− 2.95 eV). Although there is not a precise linear association between µ and IC₅₀, moderate µ values (about − 4.3 to − 4.5 eV), as found in **NA-5** and **NA-6**, appear beneficial for balancing electron donation and radical stabilization, resulting in enhanced antioxidant activity. Compound **NA-1**possesses a greater HOMO energy (− 6.11 eV) compared to compound **NA-2** (− 6.43 eV). The HOMO of compound **NA-1**is located on the 1,2,3-triazole ring and the carboxamide group, whereas the LUMO is located on the 1,3-dioxoisoindoline (phthalimide) moiety. Compound **NA-2** possesses a markedly lower LUMO (− 3.47) eV compared to compound **NA-1** (− 2.44 eV). The nitro group enhances this by stabilizing the LUMO via significant resonance withdrawal. In the case of compound **NA-2**, the delocalization of HOMO is directed towards the p-tolyl ring, while the LUMO is likely to be localized on the phthalimide moiety. Compound **NA-2** exhibits heightened reactivity, increased electrophilicity, and a decreased energy gap attributable to the potent electron-withdrawing nitro group. Compound **NA-2** displays the narrowest HOMO–LUMO energy gap (Eg = 1.17 eV), indicating high chemical reactivity. Its low HOMO energy (− 6.43 eV) reflects reduced nucleophilicity and enhanced electronic stability, while the LUMO energy (− 3.47 eV) suggests a strong electrophilic character. The HOMO is primarily localized on the triazole ring, cyano (− CN), and carbonyl (C=O) groups of the pyridone moiety, whereas the LUMO is concentrated on the pyridone and triazole rings. Additionally, **NA-4** exhibits the lowest electronegativity among the studied compounds, supporting its distinct electronic behavior.

**Fig. 3:**
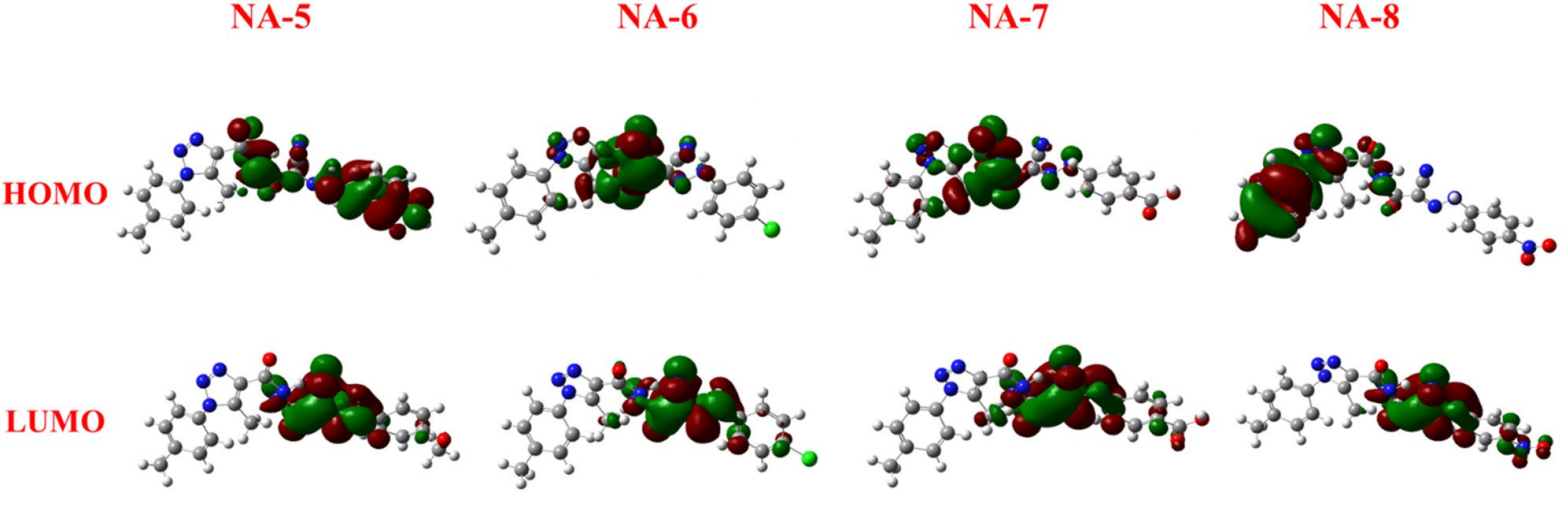
HOMOs and LUMOs geometries for compounds **NA-5-8**

As shown in Fig. 3, the presence of a methoxy group (strong electron-donating substituent) in (**NA-5**) stabilizes the HOMO and increases the energy gap, it shows Eg (3.28 eV), signifying lowing the reactivity. Compound **NA-5**, with the high hardness (η=1.64 eV), consistent with its larger HOMO–LUMO gap and has the high nucleophilicity (Nu = 0.16 eV), highlighting its potential as a strong electron donor. Compound **NA-6** has the low value of electronegativity (4.32 eV), likely due to the moderate electron-withdrawing nature of the chloro group. Among **NA-5–NA-8**,** NA-6** shows the highest softness (σ=0.45 eV^− 1^). For **NA-6** and **NA-7**, HOMO is localized on 1,2,3-triazole carbohydrazide. The compound’s **NA-7** moderate hardness (1.52 eV) and electrophilicity index (7.29 eV) further indicate its potential for effective interactions with biological nucleophiles. In compound **NA-8**, the HOMO is localized on the p-tolyl ring and the 1,2,3-triazole carbohydrazide, while the LUMO is localized on the nitro group (-NO_2_) and its aromatic ring, as well as the cyano (–C ≡ N) and carbonyl (C=O) groups. Compound **NA-8** shows the highest electronegativity (*Χ* = 5.13 eV), consistent with the presence of a nitro group, a strong electron-withdrawing moiety. Compound **NA-8** exhibits the highest electrophilicity index (ω=9.13 eV).

### Antioxidant activity

Subsequent analysis demonstrated that the newly discovered chemicals displayed antioxidant characteristics. In this phase of the inquiry, each chemical was solubilized in DMSO to assess its activity under uniform conditions. The DPPH (2,2-diphenyl-1-picrylhydrazyl) free radical scavenging test is often used to measure how well new compounds can act as antioxidants. The absorbance measurements from the test samples, in comparison to the control samples, facilitated the calculation of the percentage of antioxidant activity (Table 2) [41].

**Table 2:** Provides the results of the antioxidant evaluation for the examined compounds **(NA-1-8)**, encompassing their IC_50_ (mg/mL), percentage of residual DPPH, and percentage of free radical scavenging activity

Sample	Concentrations (mg/mL)	%Remaining DPPH	%Scavenging activity	IC_50_ (mg/mL)	
**NA-1**	0.888	56.41 ± 0.92	43.59 ± 0.92		0.996 ± 0.652
	0.444	65.31 ± 0.34	34.69 ± 0.34		
	0.222	90.63 ± 0.53	9.375 ± 0.53		
	0.111	95.47 ± 0.82	4.531 ± 0.82		
**NA-2**	0.222	20.31 ± 0.11	79.69 ± 0.11		0.072 ± 0.272
	0.111	33.91 ± 0.23	66.09 ± 0.23		
	0.055	51.56 ± 0.15	48.44 ± 0.15		
	0.028	76.56 ± 0.60	23.44 ± 0.60		
**NA-3**	2.663	26.07 ± 0.17	73.93 ± 0.17		0.830 ± 0.311
	1.331	42.42 ± 0.09	57.58 ± 0.09		
	0.666	49.89 ± 0.69	50.11 ± 0.69		
	0.333	62.44 ± 0.30	37.56 ± 0.30		
**NA-4**	0.888	56.25 ± 0.52	43.75 ± 0.52		0.989 ± 1.270
	0.444	65.63 ± 0.25	34.38 ± 0.25		
	0.222	90.63 ± 0.07	9.375 ± 0.07		
	0.111	96.56 ± 0.43	3.438 ± 0.43		
**NA-5**	0.055	31.72 ± 0.08	68.28 ± 0.08		0.029 ± 0.135
	0.028	52.81 ± 0.05	47.19 ± 0.05		
	0.014	63.13 ± 0.13	36.88 ± 0.13		
	0.007	78.13 ± 0.28	21.88 ± 0.28		
**NA-6**	0.222	20.47 ± 0.01	79.53 ± 0.01		0.096 ± 0.085
	0.111	39.84 ± 0.03	60.16 ± 0.03		
	0.055	72.03 ± 0.08	27.97 ± 0.08		
	0.028	82.81 ± 0.22	17.19 ± 0.22		
**NA-7**	0.888	60.78 ± 0.58	39.22 ± 0.58		1.256 ± 0.375
	0.444	78.44 ± 0.72	21.56 ± 0.72		
	0.222	78.91 ± 0.11	21.09 ± 0.11		
	0.111	96.88 ± 0.09	3.125 ± 0.09		
**NA-8**	0.888	50.16 ± 0.32	49.84 ± 0.32		0.839 ± 0.185
	0.444	62.66 ± 0.28	37.34 ± 0.28		
	0.222	79.69 ± 0.08	20.31 ± 0.08		
	0.111	95.16 ± 0.06	4.844 ± 0.06		
Ascorbic acid	0.06	15.27 ± 0.02	84.73 ± 0.02		0.022 ± 0.025
	0.03	39.08 ± 0.04	60.92 ± 0.04		
	0.02	61.07 ± 0.03	38.93 ± 0.03		
	0.01	74.81 ± 0.01	25.19 ± 0.01		

**Fig. 4:**
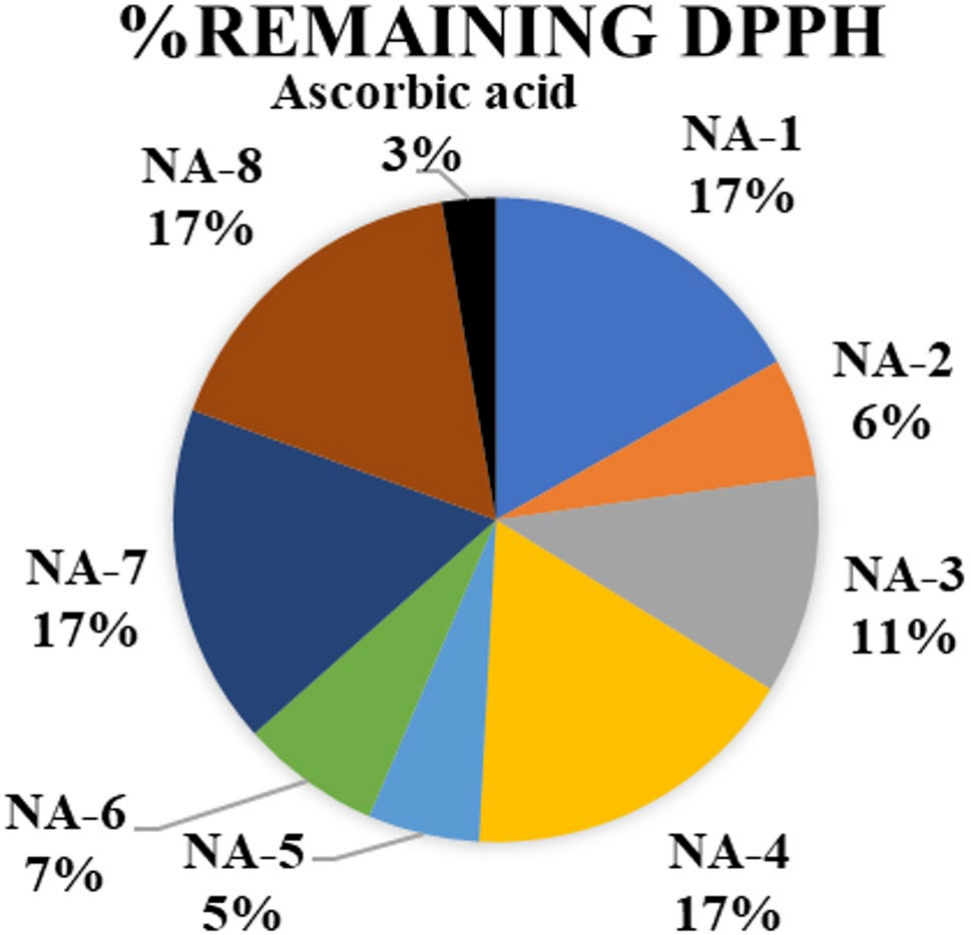
For the percentage of free radical remaining activity, dose-responsive curves were plotted

**Fig. 5:**
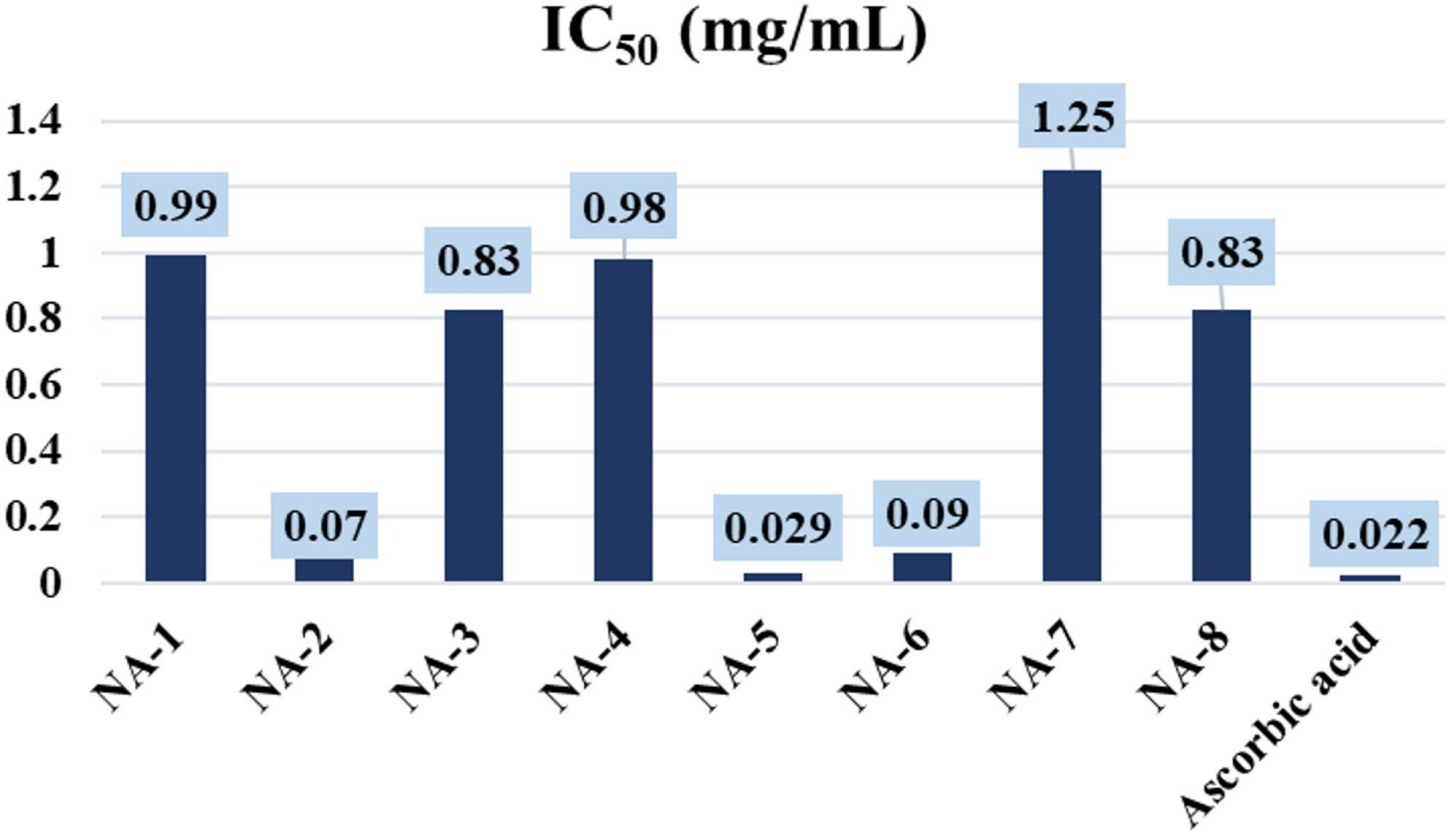
Comparison of the antioxidant results expressed as IC_50_ in mg/mL of the tested samples for (**NA-1-8**) relative to the antioxidant standard

The antioxidant activity of the newly synthesized carbohydrazide derivatives (**NA-1 to NA-8**) was assessed using the 2,2′-diphenyl-1-picrylhydrazyl (DPPH) radical scavenging assay. The assay was performed in dimethyl sulfoxide (DMSO) at a DPPH concentration of 0.135 mg/mL, using a modified protocol. Ascorbic acid was included as a standard antioxidant for comparison. All experiments were conducted in triplicate, and the average values of percentage DPPH scavenging and IC₅₀ (the concentration required to scavenge 50% of the DPPH radical) were calculated. The results are summarized in Figs. 4 and 5 and detailed in Table 2. Among all tested compounds, ascorbic acid showed the highest antioxidant activity, with the lowest residual DPPH value (15.27%) at 0.06 mg/mL and an IC_50_ of 0.022 mg/mL, confirming its role as a strong positive control.

The antioxidant activities of the synthesized carbohydrazide derivatives were evaluated using the DPPH radical scavenging assay, with IC_50_ values serving as quantitative indicators of radical scavenging efficacy. **NA-2** demonstrated strong activity (IC_50_=0.072 ± 0.272 mg/mL, 79.69% scavenging at 0.222 mg/mL), likely due to the presence of a nitro group, which can enhance electron delocalization and stabilize the resulting radical species. **NA-3** (IC_50_=0.830 ± 0.311 mg/mL) and **NA-4** (IC_50_=0.989 ± 1.270 mg/mL) displayed moderate to weak antioxidant activities. Among the tested compounds, **NA-5** exhibited the highest antioxidant activity, with an IC_50_ value of 0.029 ± 0.135 mg/mL, comparable to that of ascorbic acid (0.022 ± 0.025 mg/mL). This enhanced activity is attributed to the presence of an electron-donating 4-methoxy group, which contributes to radical stabilization through resonance effects. **NA-6** showed moderate-to-high antioxidant activity (IC_50_=0.096 ± 0.085 mg/mL, 79.53% scavenging at 0.222 mg/mL), potentially influenced by the 4-chlorophenyl substituent, which exerts an inductive electron-withdrawing effect. **NA-7** showed the lowest antioxidant activity among all derivatives, with an IC_50_ of 1.256 ± 0.375 mg/mL and only 3.13% scavenging at 0.111 mg/mL. The reduced activity of **NA-7** is likely due to steric hindrance and the presence of an electron-withdrawing carboxylic acid group, which decreases electron density at the radical-reactive site. Compounds **NA-1** (IC_50_=0.996 ± 0.652 mg/mL) and **NA-8** (IC_50_=0.839 ± 0.185 mg/mL) exhibited limited antioxidant activity.

Based on IC_50_ values, the compounds were ranked in descending order of antioxidant activity as follows:

**Ascorbic acid < NA-5 < NA-2 < NA-6 < NA-3 < NA-8 < NA-4 < NA-1 < NA-7.**

### Pharmacokinetic parameter estimation

The SwissADME platform was employed for the in silico ADME (Absorption, Distribution, Metabolism, and Excretion) evaluation of candidate compounds, offering critical insights into their pharmacokinetic profiles. The analysis was based on key physicochemical descriptors such as rotatable bonds, topological polar surface area (TPSA), hydrogen bond donors and acceptors, and molecular weight Table 3. These parameters enabled robust predictions regarding intestinal absorption, blood–brain barrier (BBB) penetration, and membrane permeability, collectively informing the compounds’ potential oral bioavailability, CNS accessibility, and overall drug-likeness.

A comprehensive summary of the in silico ADMET predictions, including molecular structures, physicochemical properties, solubility profiles, gastrointestinal absorption, metabolic interactions, and drug-likeness parameters of compounds (**1**) and **NA-1–8**, is provided in Tables S1–S7 in the Supplementary File.

Such predictive models are essential in the early stages of drug discovery, where they serve to prioritize compounds with favorable pharmacokinetics and eliminate those with suboptimal profiles. Ultimately, this computational strategy supports a more focused, time-efficient, and cost-effective approach to subsequent in vitro and in vivo experimental validation. Molar Refractivity (MR) is a physicochemical parameter that reflects the total polarizability of a molecule, incorporating both electronic and steric contributions [42]. It is derived from the Lorentz–Lorenz equation [43] and is mathematically related to a molecule’s volume and refractive index. A higher MR indicates a larger or more polarizable molecule, often associated with greater capacity for radical stabilization, a key feature of antioxidant compounds. In the current study, MR values ranged from 62.19 (**Compound 1**) to 117.99 (**NA-8**), illustrating significant variation in molecular size and electronic properties. For instance, **Compound 1** (MR = 62.19) is less polarizable than **NA-1** (MR = 99.28), which may influence both reactivity and membrane interaction. Topological Polar Surface Area (TPSA) is a two-dimensional molecular descriptor that quantifies the surface area occupied by polar atoms—primarily oxygen and nitrogen—and their associated hydrogen atoms [44]. Unlike full 3D surface calculations, TPSA employs fragment-based topological contributions, allowing for rapid estimation of molecular polarity and its impact on permeability and BBB penetration [45]. Compounds with TPSA < 140 Å² are generally associated with favorable GI absorption and CNS penetration, while those with TPSA > 140 Å² are likely to exhibit poor membrane permeability. For example, **Compound 1** (TPSA = 85.83) is well-positioned for both GI and BBB absorption. In contrast, **NA-2** (TPSA = 143.01) and **NA-5** (TPSA=146.32) are predicted to have reduced oral bioavailability due to excessive polarity. Hydrogen bonding potential, defined by the number of hydrogen bond donors and acceptors, plays a crucial role in target binding affinity and specificity. Compounds with high hydrogen bonding capacity are more likely to engage in favorable interactions with protein binding sites, enhancing biological activity. Molecular flexibility, assessed through the number of rotatable bonds, also contributes to binding efficacy by enabling better conformational adaptation to receptor geometries. Compounds such as **NA-5**, **NA-7**, and **NA-8** demonstrate greater flexibility, which may improve receptor interaction. Lipophilicity, often expressed as Log P, is another essential parameter affecting membrane permeability, receptor binding within hydrophobic environments, and metabolic stability. The SwissADME platform (http://www.swissadme.ch/) was employed to evaluate pharmacokinetic and physicochemical properties using a combination of 27 molecular descriptors and topological features, some of which are derived from tools such as SILICOS-IT [46]. A key output is the consensus Log Po/w, calculated as the average of five distinct prediction models. Most compounds displayed Log P values below 5, aligning with Lipinski’s rule of five, and indicating good oral bioavailability and lipophilic compatibility. In silico predictions also suggested that the ranged from soluble to poorly soluble depending on the analog, supporting formulation flexibility and efficient physiological absorption. However, several compounds may be subject to active transport or efflux across biological barriers such as the intestinal wall, BBB, or renal/biliary systems. These effects are often mediated by efflux transporters, particularly P-glycoprotein (P-gp), a member of the ATP-binding cassette (ABC) transporter family. Its expression in the GI tract plays a critical role in drug absorption, and compounds that are substrates of P-gp may experience reduced cellular uptake due to active efflux mechanisms. The bioactivity score is a computational estimate of a compound’s potential to exhibit biological activity in vivo. Scores above 0 are typically indicative of likely therapeutic efficacy. All compounds in this study demonstrated bioactivity scores of approximately 0.55, suggesting a high probability of relevant biological effects in clinical or preclinical contexts. The data highlight the importance of integrating electronic properties (high MR), polar surface considerations (moderate TPSA), and pharmacokinetic behavior (high GI absorption) to identify optimal antioxidant candidates.

**Table 3:** ADME and drug-likeness profiles of compounds **1** and **NA-1-8**

Molecule	Rotatable bonds	H-bond acceptors	H-bond donors	MR	TPSA	MLOGP	ESOL solubility (mol/l)	GI absorption	BBB permeant	Pgp substrate	Lipinski violations	PAINS alerts
**1**	3	4	2	62.19	85.83	1.17	6.73E-03	High	No	No	0	0
**NA-1**	4	5	1	99.28	97.19	2.59	1.22E-04	High	No	No	0	0
**NA-2**	5	7	1	108.1	143.01	1.5	1.06E-04	Low	No	Yes	1	0
**NA-3**	6	5	2	76.65	112.7	0.65	4.28E-03	High	No	No	0	0
**NA-4**	4	5	1	100.15	105.6	2.32	1.83E-04	High	No	No	0	0
**NA-5**	9	7	3	115.66	146.32	1.07	1.58E-05	Low	No	No	1	1
**NA-6**	8	6	3	114.17	137.09	1.83	4.74E-06	High	No	No	0	1
**NA-7**	9	8	4	116.12	174.39	-0.05	2.51E-05	Low	No	Yes	1	1
**NA-8**	9	8	3	117.99	182.91	0.56	1.60E-05	Low	No	Yes	1	1

## Conclusion

A number of compounds based on carbohydrazide’s (**NA-1 to NA-8**) were successfully synthesized by the use of effective techniques. 5-methyl-1-(p-tolyl)-1*H*-1,2,3-triazole-4-carbohydrazide **(1)** was the starting point for several reactions that produced the target compounds in good to high yields using phthalic anhydride derivatives (**2a**,**2b**), acetylacetone (**4**), and different diazonium (**5a-d**) salts. The structures, which displayed the anticipated functional groups and molecular weights, were validated by a number of methods, including mass spectrometry, NMR, and infrared. These outcomes show how well the selected synthetic pathways work to provide a range of functionalized triazole carbohydrazide derivatives (**NA-1-8**) with potential uses in other fields. The reactivity, stability, and electronic behavior of carbohydrazide derivatives (**NA-1 to NA-8**) were examined using frontier molecular orbital (FMO) analysis, which demonstrated the effects of various substituents. Lower energy gaps and more electrophilicity were seen in compounds having electron-withdrawing groups such as nitro (**NA-2** and **NA-8**), suggesting increased reactivity. On the other hand, compounds having methoxy and other electron-donating groups as (**NA-5**) exhibited stronger nucleophilicity and larger energy gaps, indicating lesser reactivity and more stability. Using the DPPH technique, the antioxidant activity of the produced carbohydrazide compounds (**NA-1 to NA-8**) was assessed. Compounds **NA-2**, **NA-5**, and **NA-6** all exhibited good to strong antioxidant activity, **NA-5** functioned nearly as well as ascorbic acid, the common antioxidant. On the other hand, compounds **NA-1**,** NA-4**,** NA-7**, and **NA-8** exhibited relatively low levels of antioxidant activity, most likely as a result of less advantageous structural characteristics such bulky or electron-withdrawing groups. Overall, the findings indicate that these compounds’ antioxidant activity is significantly influenced by substituents on their aromatic rings. SwissADME was used for the in silico ADME investigation, which demonstrated the good drug-like characteristics of the synthesized carbohydrazide compounds. Certain adaptable substances, such as **NA-5**,** NA-7**, and **NA-8**, might suit target receptors more precisely. Additionally, the compounds demonstrated good water solubility, which is crucial for medication formulation and absorption. Potential impacts on the medications’ absorption or distribution are suggested by anticipated interactions with biological transporters such as P-glycoprotein. With bioactivity scores of approximately 0.55, all substances showed promise for biological activity and probable success in upcoming clinical trials. All things considered, these substances show promise as potential drugs.

## Supplementary Information

Below is the link to the electronic supplementary material.

Supplementary Material 1

## Data Availability

The datasets used and/or analysed during the current study are available from the corresponding author on reasonable request.
