# Supplementary material for: Design, synthesis, and evaluation of novel triazole-based carbohydrazide derivatives with notable antioxidant activity: an integrated experimental and DFT study
Source: BMC Chem. 2026 Feb 23;20(1):48. doi: 10.1186/s13065-026-01736-x (PMC12955047; doi:10.1186/s13065-026-01736-x)
Supplement: Supplementary file 1 — Supplementary Material 1 [file 13065_2026_1736_MOESM1_ESM.docx]

**Table S1. Pharmaceutical prediction of in silico ADMET properties of compounds (1) and NA-1-8.**

| code | Smile mode | Formula | MW |
| --- | --- | --- | --- |
| 1 | CC1=CC=C(N2C(C)=C(C(NN)=O)N=N2)C=C1 | **C_11_H_13_N_5_O** | 231.26 |
| NA-1 | CC1=CC=C(N2C(C)=C(C(NN3C(C(C=CC=C4)=C4C3=O)=O)=O)N=N2)C=C1 | **C_19_H_15_N_5_O_3_** | 361.36 |
| NA-2 | CC1=CC=C(N2C(C)=C(C(NN3C(C(C=C([N+]([O-])=O)C=C4)=C4C3=O)=O)=O)N=N2)C=C1 | **C_19_H_14_N_6_O_5_** | 406.36 |
| NA-3 | O=C(NNC(C(N=NN1C(C=C2)=CC=C2C)=C1C)=O)CC#N | **C_14_H_14_N_6_O_2_** | 298.31 |
| NA-4 | CC1=CC=C(N2C(C)=C(C(NN3C(C(C#N)=C(C)C=C3C)=O)=O)N=N2)C=C1 | **C_19_H_18_N_6_O_2_** | 362.39 |
| NA-5 | O=C(NNC(C(N=NN1C(C=C2)=CC=C2C)=C1C)=O)/C(C#N)=N/NC3=CC=C(OC)C=C3 | **C_21_H_20_N_8_O_3_** | 432.44 |
| NA-6 | O=C(NNC(C(N=NN1C(C=C2)=CC=C2C)=C1C)=O)/C(C#N)=N/NC3=CC=C(Cl)C=C3 | **C_20_H_17_ClN_8_O_2_** | 436.86 |
| NA-7 | O=C(NNC(C(N=NN1C(C=C2)=CC=C2C)=C1C)=O)/C(C#N)=N/NC3=CC=C(C(O)=O)C=C3 | **C_21_H_18_N_8_O_4_** | 446.43 |
| NA-8 | O=C(NNC(C(N=NN1C(C=C2)=CC=C2C)=C1C)=O)/C(C#N)=N/NC3=CC=C([N+]([O-])=O)C=C3 | **C_20_H_17_N_9_O_4_** | 447.42 |

**Table S2. Pharmaceutical prediction of in silico ADMET properties of compounds (1) and NA-1-8.**

| Molecule | MR | TPSA | iLOGP | XLOGP3 | WLOGP | MLOGP | Silicos-IT Log P | Consensus Log P | ESOL Log S |
| --- | --- | --- | --- | --- | --- | --- | --- | --- | --- |
| 1 | 62.19 | 85.83 | 2.06 | 0.98 | 0.49 | 1.17 | 0.26 | 0.99 | -2.17 |
| NA-1 | 99.28 | 97.19 | 2.6 | 2.59 | 1.44 | 2.59 | 1.54 | 2.15 | -3.91 |
| NA-2 | 108.1 | 143.01 | 2.13 | 2.42 | 1.35 | 1.5 | -0.61 | 1.36 | -3.97 |
| NA-3 | 76.65 | 112.7 | 1.51 | 1.12 | 0.56 | 0.65 | 0.51 | 0.87 | -2.37 |
| NA-4 | 100.15 | 105.6 | 2.88 | 2.3 | 1.73 | 2.32 | 2.16 | 2.28 | -3.74 |
| NA-5 | 115.66 | 146.32 | 2.51 | 3.94 | 1.45 | 1.07 | 1.17 | 2.03 | -4.8 |
| NA-6 | 114.17 | 137.09 | 2.9 | 4.6 | 2.1 | 1.83 | 1.74 | 2.63 | -5.32 |
| NA-7 | 116.12 | 174.39 | 1.61 | 3.5 | 1.14 | -0.05 | 0.58 | 1.36 | -4.6 |
| NA-8 | 117.99 | 182.91 | 1.86 | 3.8 | 1.35 | 0.56 | -1.03 | 1.31 | -4.8 |

**Table S3. Pharmaceutical prediction of in silico ADMET properties of compounds (1) and NA-1-8.**

| Molecule | ESOL Solubility (mg/ml) | ESOL Solubility (mol/l) | ESOL Class | Ali Log S | Ali Solubility (mg/ml) | Ali Solubility (mol/l) |
| --- | --- | --- | --- | --- | --- | --- |
| 1 | 1.56E+00 | 6.73E-03 | Soluble | -2.37 | 9.85E-01 | 4.26E-03 |
| NA-1 | 4.40E-02 | 1.22E-04 | Soluble | -4.28 | 1.90E-02 | 5.25E-05 |
| NA-2 | 4.32E-02 | 1.06E-04 | Soluble | -5.07 | 3.49E-03 | 8.60E-06 |
| NA-3 | 1.28E+00 | 4.28E-03 | Soluble | -3.08 | 2.48E-01 | 8.32E-04 |
| NA-4 | 6.63E-02 | 1.83E-04 | Soluble | -4.16 | 2.53E-02 | 6.99E-05 |
| NA-5 | 6.82E-03 | 1.58E-05 | Moderately soluble | -6.71 | 8.38E-05 | 1.94E-07 |
| NA-6 | 2.07E-03 | 4.74E-06 | Moderately soluble | -7.2 | 2.73E-05 | 6.26E-08 |
| NA-7 | 1.12E-02 | 2.51E-05 | Moderately soluble | -6.85 | 6.37E-05 | 1.43E-07 |
| NA-8 | 7.17E-03 | 1.60E-05 | Moderately soluble | -7.34 | 2.07E-05 | 4.62E-08 |

**Table S4. Pharmaceutical prediction of in silico ADMET properties of compounds (1) and NA-1-8.**

| Molecule | Ali Class | Silicos-IT LogSw | Silicos-IT Solubility (mg/ml) | Silicos-IT Solubility (mol/l) | Silicos-IT class | GI absorption |
| --- | --- | --- | --- | --- | --- | --- |
| 1 | Soluble | -2.98 | 2.43E-01 | 1.05E-03 | Soluble | High |
| NA-1 | Moderately soluble | -5.46 | 1.24E-03 | 3.44E-06 | Moderately soluble | High |
| NA-2 | Moderately soluble | -4.81 | 6.32E-03 | 1.56E-05 | Moderately soluble | Low |
| NA-3 | Soluble | -3.81 | 4.61E-02 | 1.55E-04 | Soluble | High |
| NA-4 | Moderately soluble | -5.37 | 1.55E-03 | 4.28E-06 | Moderately soluble | High |
| NA-5 | Poorly soluble | -6.1 | 3.44E-04 | 7.96E-07 | Poorly soluble | Low |
| NA-6 | Poorly soluble | -6.58 | 1.14E-04 | 2.61E-07 | Poorly soluble | High |
| NA-7 | Poorly soluble | -5.33 | 2.08E-03 | 4.67E-06 | Moderately soluble | Low |
| NA-8 | Poorly soluble | -5.34 | 2.06E-03 | 4.61E-06 | Moderately soluble | Low |

**Table S5. Pharmaceutical prediction of in silico ADMET properties of compounds (1) and NA-1-8.**

| Molecule | BBB permeant | Pgp substrate | CYP1A2 inhibitor | CYP2C19 inhibitor | CYP2C9 inhibitor | CYP2D6 inhibitor |
| --- | --- | --- | --- | --- | --- | --- |
| 1 | No | No | Yes | No | No | No |
| NA-1 | No | No | No | Yes | Yes | No |
| NA-2 | No | Yes | No | No | Yes | No |
| NA-3 | No | No | No | No | No | No |
| NA-4 | No | No | No | No | Yes | No |
| NA-5 | No | No | No | No | Yes | No |
| NA-6 | No | No | No | No | Yes | No |
| NA-7 | No | Yes | No | No | No | No |
| NA-8 | No | Yes | No | No | Yes | No |

**Table S6. Pharmaceutical prediction of in silico ADMET properties of compounds (1) and NA-1-8.**

| Molecule | CYP3A4 inhibitor | log Kp (cm/s) | Lipinski #violations | Ghose #violations | Veber #violations | Egan #violations |
| --- | --- | --- | --- | --- | --- | --- |
| 1 | No | -7.01 | 0 | 0 | 0 | 0 |
| NA-1 | No | -6.67 | 0 | 0 | 0 | 0 |
| NA-2 | No | -7.06 | 1 | 0 | 1 | 1 |
| NA-3 | No | -7.32 | 0 | 0 | 0 | 0 |
| NA-4 | No | -6.88 | 0 | 0 | 0 | 0 |
| NA-5 | Yes | -6.14 | 1 | 0 | 1 | 1 |
| NA-6 | Yes | -5.7 | 0 | 0 | 0 | 1 |
| NA-7 | Yes | -6.54 | 1 | 0 | 1 | 1 |
| NA-8 | Yes | -6.33 | 1 | 0 | 1 | 1 |

**Table S7. Pharmaceutical prediction of in silico ADMET properties of compounds (1) and NA-1-8.**

| Molecule | Muegge #violations | Bioavailability Score | PAINS #alerts | Brenk #alerts | Leadlikeness #violations | Synthetic Accessibility |
| --- | --- | --- | --- | --- | --- | --- |
| **1** | **0** | **0.55** | **0** | **2** | **1** | **2.45** |
| **NA-1** | **0** | **0.55** | **0** | **1** | **1** | **3.03** |
| **NA-2** | **0** | **0.55** | **0** | **3** | **1** | **3.22** |
| **NA-3** | **0** | **0.55** | **0** | **0** | **0** | **2.92** |
| **NA-4** | **0** | **0.55** | **0** | **0** | **1** | **3.35** |
| **NA-5** | **0** | **0.55** | **1** | **1** | **3** | **3.78** |
| **NA-6** | **0** | **0.55** | **1** | **1** | **3** | **3.69** |
| **NA-7** | **1** | **0.11** | **1** | **1** | **2** | **3.8** |
| **NA-8** | **1** | **0.55** | **1** | **3** | **3** | **3.81** |

**Fig (S1): IR spectrum of compound NA-1.**

**Fig (S2): ^1^H NMR spectrum of compound NA-1.**

**Fig (S3): ^13^C NMR spectrum of compound NA-1.**

**Fig (S4): Mass spectroscopy of compound NA-1.**

**Fig (S5): IR spectrum of compound NA-2.**

**Fig (S6): ^1^H NMR spectrum of compound NA-2.**

**Fig (S7): Mass spectroscopy of compound NA-2.**

**Fig (S8): IR spectrum of compound NA-3.**


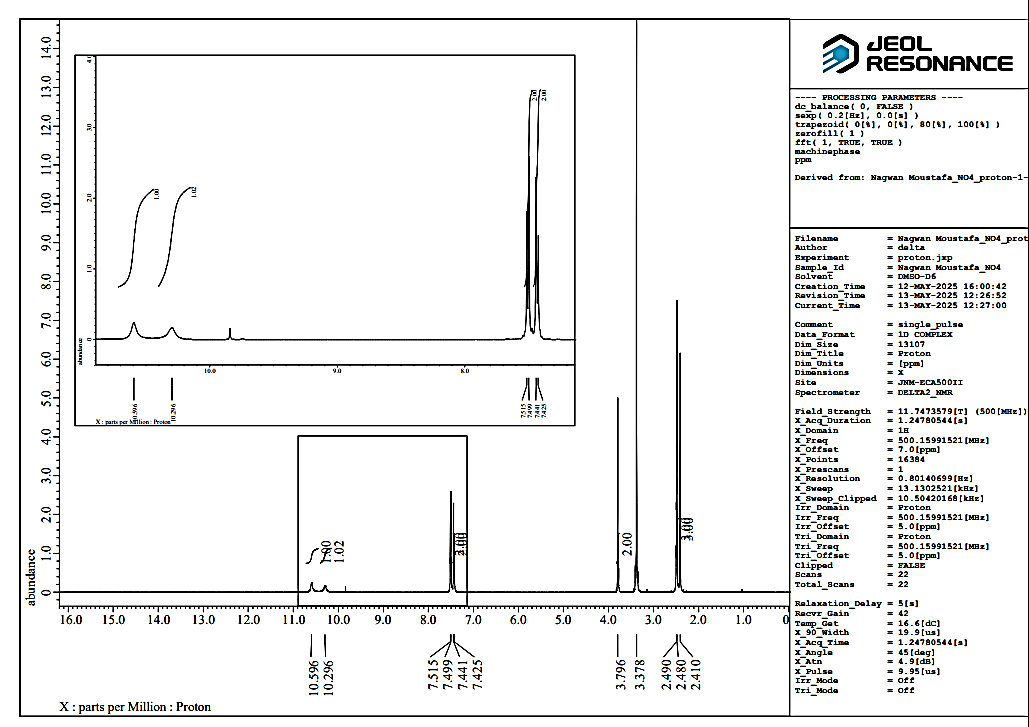

**Fig (S9): ^1^H NMR spectrum of compound NA-3.**


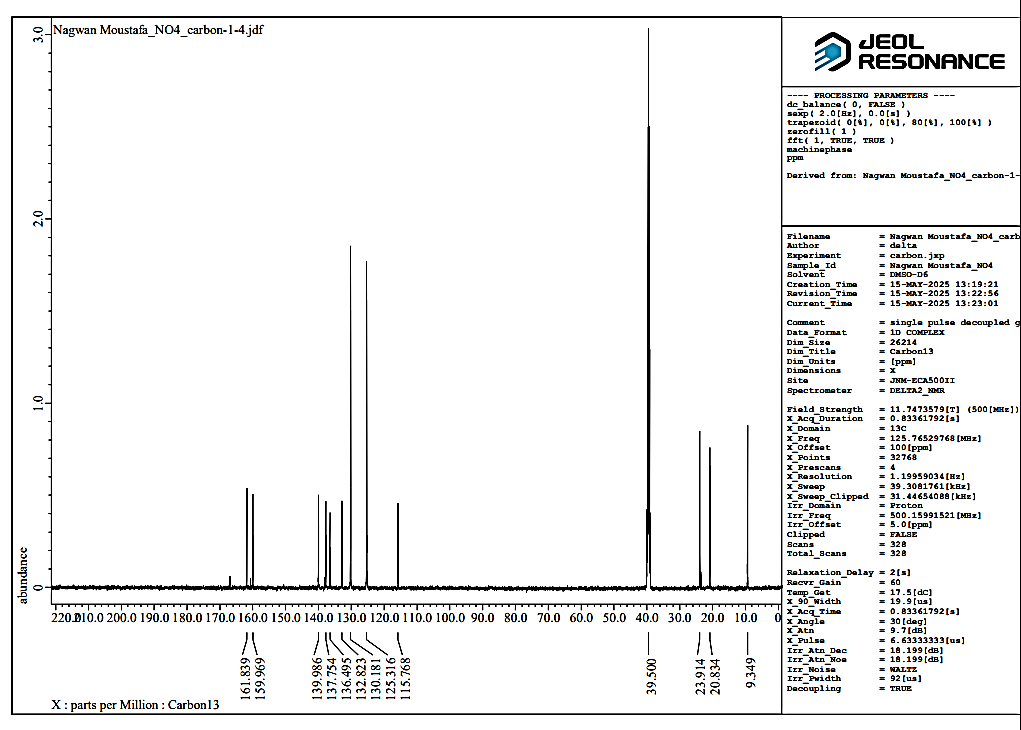

**Fig (S10): ^13^C NMR spectrum of compound NA-3.**

**Fig (S11): Mass spectroscopy of compound NA-3.**

**Fig (S12): IR spectrum of compound NA-4.**

**Fig(S13): ^1^H NMR spectrum of compound NA-4.**

**Fig(S14): ^13^C NMR spectrum of compound NA-4.**

**Fig (S15): Mass spectroscopy of compound NA-4.**

**Fig (S16): IR spectrum of compound NA-5.**


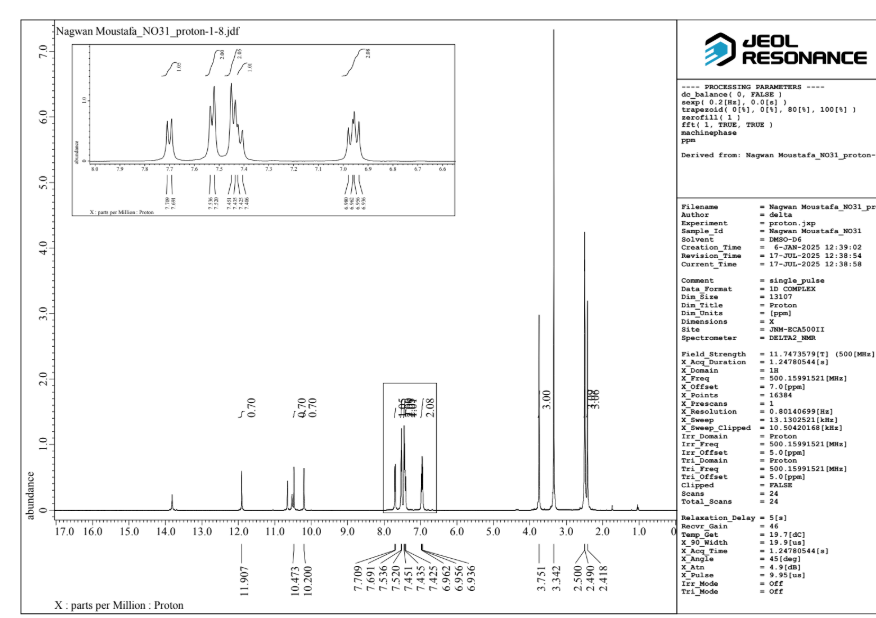

**Fig(S17): ^1^H NMR spectrum of compound NA-5.**


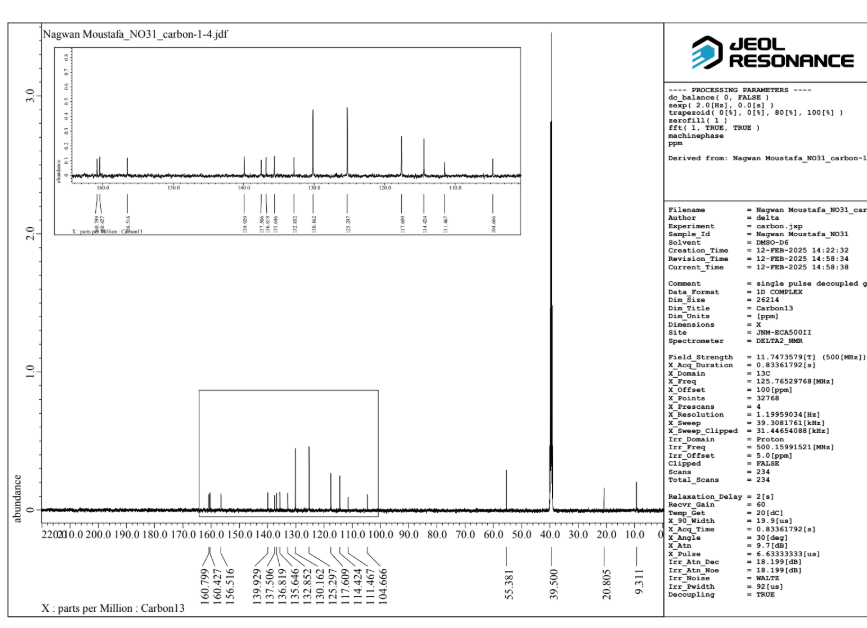

**Fig(S18): ^13^C NMR spectrum of compound NA-5.**

**Fig (S19): Mass spectroscopy of compound NA-5.**

**Fig (S20): IR spectrum of compound NA-6.**


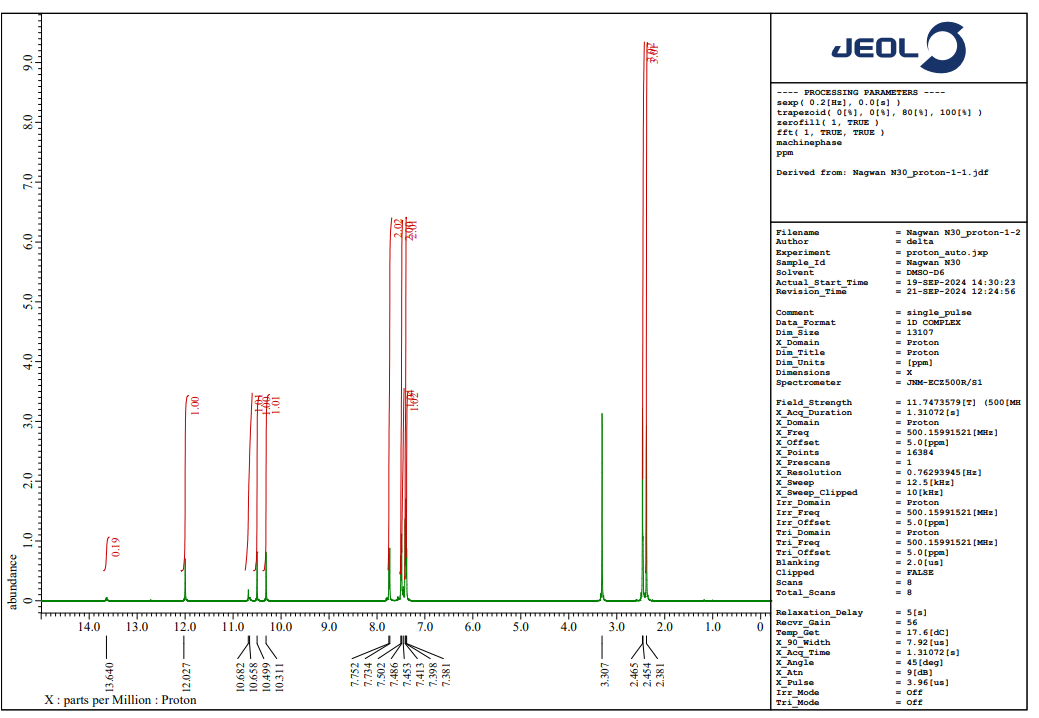

**Fig(S21): ^1^H NMR spectrum of compound NA-6.**


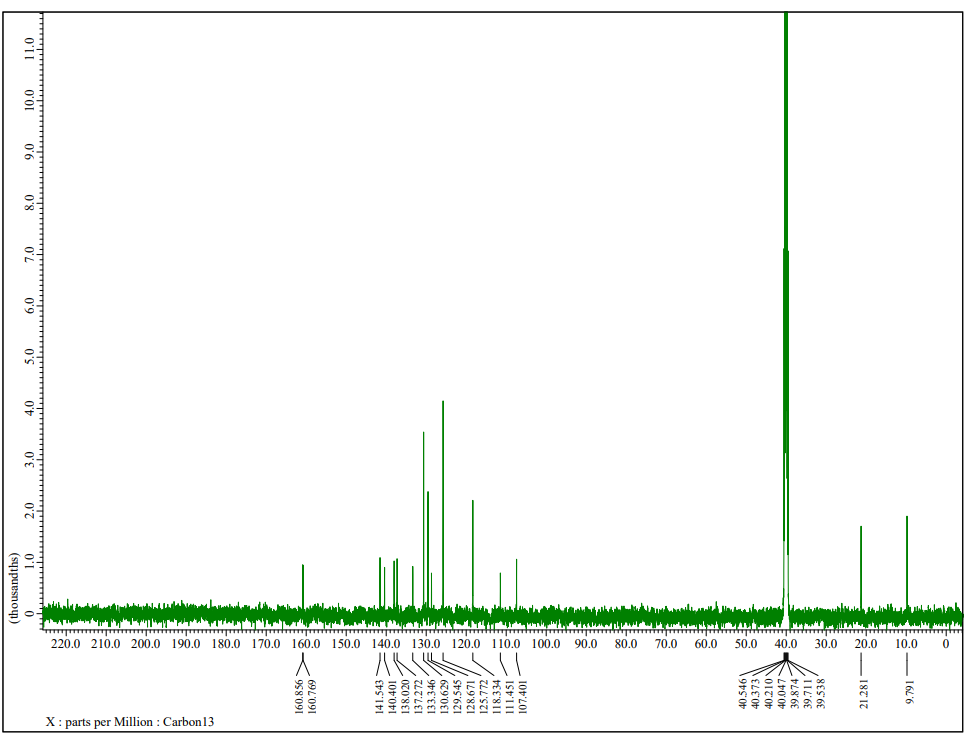

**Fig(S22): ^13^C NMR spectrum of compound NA-6.**

**Fig (S23): Mass spectroscopy of compound NA-6.**

**Fig (S24): IR spectrum of compound NA-7.**

**Fig(S25): ^1^H NMR spectrum of compound NA-7.**

**Fig(S26): ^13^C NMR spectrum of compound NA-7.**

**Fig (S27): Mass spectroscopy of compound NA-7.**

**Fig (S28): IR spectrum of compound NA-8.**

**Fig(S29): ^1^H NMR spectrum of compound NA-8.**

**Fig (S30): Mass spectroscopy of compound NA-8.**
